# Non-Invasive electrophysiological monitoring of cardiac organoids using 3D-Net-assisted microelectrodes array platform

**DOI:** 10.1038/s41598-025-34504-3

**Published:** 2026-01-24

**Authors:** Shinhye Park, Sang-Jun Cho, C-Yoon Kim, Hyung Min Chung, Seul-Gi Lee

**Affiliations:** 1 College of Veterinary Medicine, Konkuk University, Seoul, 05029 Republic of Korea; 2 Cellames Inc, 405, 19 Wiryegwangjang‑ro, Sujeong‑gu, Seongnam‑si, Gyeonggi‑do Republic of Korea; 3 Department of Stem Cell Biology, School of Medicine, Konkuk University, 120 Neungdong- Ro, Gwangjin-Gu, Seoul, 05029 Republic of Korea

**Keywords:** Cardiac organoid, 3D-Net, Electrophysiological monitoring, Cardiotoxicity, Microelectode array, Biological techniques, Biotechnology, Engineering, Medical research

## Abstract

The rapid advancement of organoids has increased interest in replacing conventional 2D alternatives to animal testing. In drug development, cardiotoxicity assessment is essential, and electrophysiological signals from cardiomyocytes serve as key biomarkers. Accordingly, planar 2D microelectrode array (MEA) chips are widely used. However, cardiomyocytes-based platform fails to reproduce complex in vivo physiological responses, leading to the combination of human cardiac organoids (hCdOs) with 3D sensors. Most of these approaches remain in the research stage and lack standardization. Therefore, there is still a need for methods that can stably evaluate organoids using commercially available 2D MEA chips. In this study, a silicon-based 3D-Net was introduced to enable stable monitoring of hCdOs electrophysiological signals on MEA chip. hCdOs fabricated using established protocols showed morphological and functional reproducibility suitable for drug testing. The 3D-Net prevented organoid flotation, ensured sufficient medium supply, and enabled measurements beyond acute assessment periods without functional impairment. Drug responsiveness varied significantly depending on the presence of 3D-Net and the volume of drug-containing medium. The 3D-Net platform overcomes the limitations of MEA, providing an effective and non-invasive method for functional evaluation and drug screening of hCdOs. Furthermore, this approach is expected to support preclinical evaluation of drug efficacy and safety.

**Supplementary Information:**

The online version contains supplementary material available at 10.1038/s41598-025-34504-3.

## Introduction

Cardiotoxicity assessment is a critical component of the preclinical stage in drug development^1^. The recent enactment of the FDA Modernization Act 2.0 has further underscored the importance of alternatives to animal testing^2^. Among cardiotoxicity indicators, QT interval prolongation serves as an important predictor of potential arrhythmia, and 2D electrophysiological analysis platforms based on human induced pluripotent stem cell–derived cardiomyocytes (hiPSC-CMs) are widely utilized for this purpose^3–6^. Owing to their high reproducibility and suitability for quantitative analysis, these models have become the industrial standard worldwide. However, the heart comprises multiple cell types that interact within a 3D microenvironment to regulate function^7^. This can lead to the limitation that CM-only models do not sufficiently reflect in vivo characteristics. In this context, 3D models such as hiPSC derived cardiac organoids (hCdOs) have recently garnered increasing attention^7–10^. hCdO faithfully recapitulates native cardiac architecture and cellular diversity, making it a promising platform to complement or replace 2D systems in cardiotoxicity assessment.

Various approaches have been employed to construct 3D cardiac models. Some methods use extracellular matrix (ECM) components to induce iPSCs to spontaneously differentiate into cardiac lineages, generating organoids^8,11^. Other strategies involve mixing terminally differentiated cardiac cells into hydrogels to create engineered heart tissues or applying 3D bioprinting technologies to fabricate heart-on-chips^12^. Although each model has advantages and limitations, organoids provide unique strengths. Because they form through self-organization from iPSCs, they do not have artificially imposed cellular arrangements, and multiple cardiac cells assemble to more faithfully recapitulate in vivo cardiac structure and function^8–10^. In in vitro studies, the maturation of CMs was promoted in cardiac spheroids containing cardiac fibroblasts rather than fibroblasts from other tissues^13^. Moreover, even when certain disease phenotypes emerged, their manifestation was more pronounced in co-cultures of CMs and cardiac fibroblasts^14^, highlighting the importance of cell–cell interactions. Organoids are being applied not only in drug screening and disease modeling but also in clinical trials for patient-specific therapeutic development. Therefore, as the scalability of organoids is ensured, systematic database construction and precise analyses become increasingly necessary to guarantee the reproducibility of organoid production and analysis^11,15^.

Most commercially available electrophysiological signal analysis devices, such as microelectrode array (MEA), feature planar electrode structures optimized for 2D single-cell models^5^. Such configurations pose multiple difficulties for the analysis of 3D organoids. Because organoids float in culture media or exhibit spontaneous movement, maintaining stable contact with electrodes is difficult, often resulting in signal distortion or measurement failure^8,16^. Minimizing the culture medium volume can alleviate this issue but leads to medium evaporation and instability of drug doses during prolonged monitoring. Attaching organoids to surfaces using ECM can alter cellular architecture and impair electrophysiological signals, thereby limiting quantitative analysis. Although micro-needle, shell-type, or flexible 3D sensor platforms have been explored, these remain in the research stage and have not yet been validated as standardized alternative testing methods^16–18^. Consequently, most cardiotoxicity evaluations continue to rely on established 2D system platforms^8,19,20^.

In this study, a 3D-Net system is proposed that preserves the advantages of conventional 2D platforms while enabling stable immobilization of hCdOs without damage, allowing signal analysis under sufficient culture medium conditions. A protocol for generating hCdOs suitable for drug evaluation was established, and the reproducibility of morphological and functional indices was validated^8^. Functional characteristics were compared between organoids with and without 3D-Net application, and changes in field potential were analyzed following treatment with various drugs under 3D-Net condition. This approach enables stable and quantitative drug response assessment in hCdOs on a standardized 2D platform, forming a basis for future 3D cardiotoxicity platforms.

## Results

### Characterization of hiPSC derived cardiac organoids

**Fig. 1:**
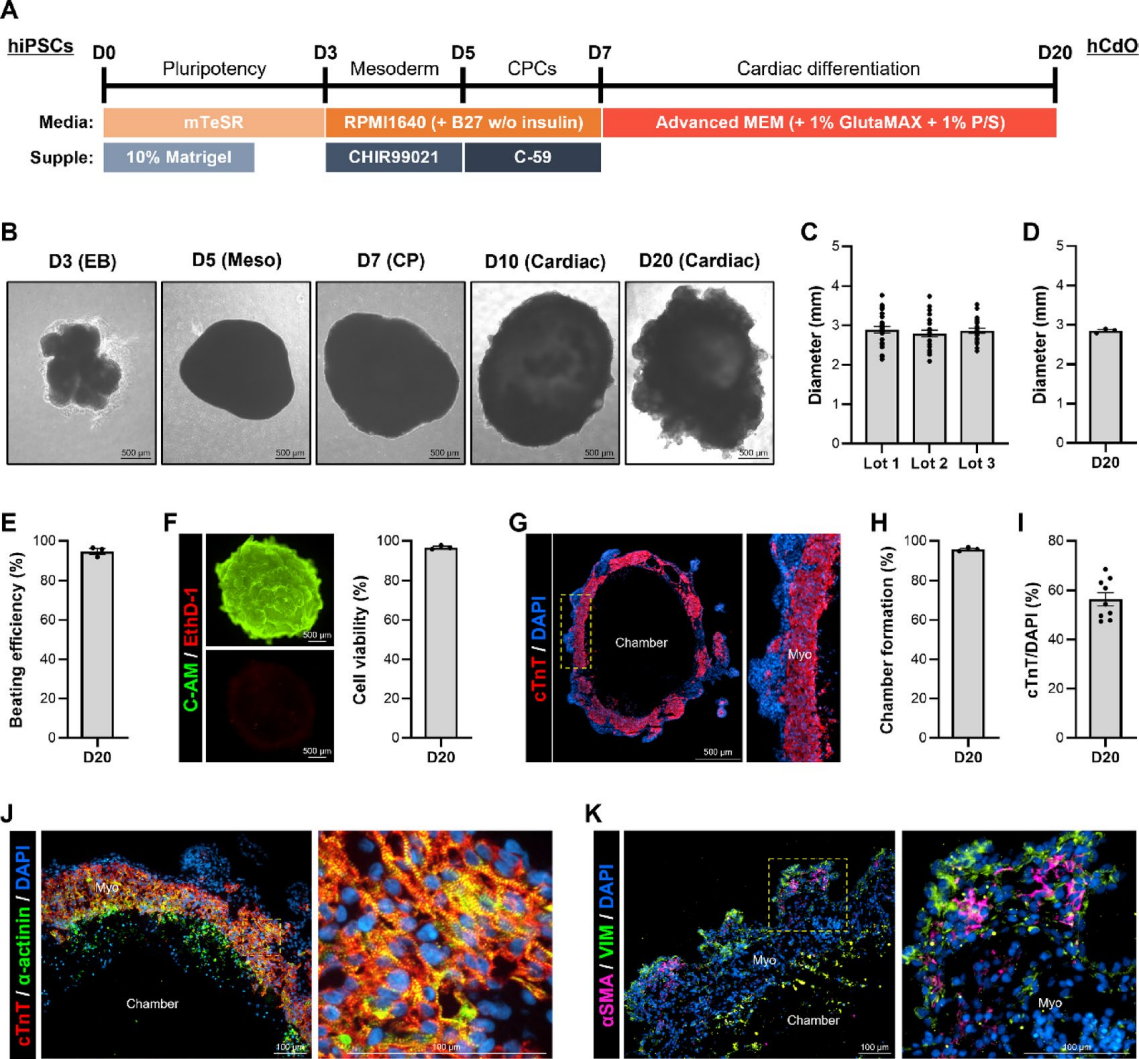
Characterization of hCdOs.(**A**) Protocol for generating hCdOs from hiPSCs. (**B**) Morphology of hCdOs at each differentiation stage. Scale bar: 500 μm. (**C**) Diameter of D20 hCdOs derived from different lots (*N* = 3, *n* = 25) and (**D**) average diameter (*N* = 3). (**E**) Beating efficiency of D20 hCdOs (*N* = 3). (**F**) Cell viability of D20 hCdOs analyzed by live (C-AM)/dead (EthD-1) assay (*N* = 3). Scale bar: 500 μm. (**G**) Identification of chamber and myocardium (Myo) in hCdO via cTnT staining. Scale bar: 500 μm. (**H**) Chamber formation (*N* = 3) and (**I**) cTnT/DAPI distribution (*n* = 9) in D20 hCdOs. (J) Confirmation of sarcomeric structures via co-staining with cTnT and α-actinin. Scale bar: 100 μm. (K) Confirmation of cardiac cells via co-staining with αSMA and VIM. Scale bar: 100 μm. Yellow dotted boxes: enlarged images. Data are expressed as the mean ± SEM.

Cardiac organoids (hCdOs) derived from human induced pluripotent stem cells (hiPSCs) were generated using a chemically defined differentiation protocol (Fig. 1A)^8^. As differentiation progressed through the stages of embryoid bodies (EB), mesoderm (Meso), cardiac progenitor (CP), and cardiac lineage, morphological changes in the hCdOs were observed (Fig. 1B). The diameters of D20 hCdOs were consistent across three different lots (Fig. 1C), with an average diameter of 2.85 ± 0.03 mm (Fig. 1D). hCdOs from different lots exhibited high functional consistency, showing a beating efficiency of 94.89 ± 1.43% and a cell viability of 96.86 ± 0.52% (Fig. 1E and F). To investigate the cardiac tissue organization within D20 hCdOs, staining for the cardiomyocyte (CM)-specific marker cardiac Troponin T (cTnT) was performed. cTnT was expressed in a belt-like pattern surrounding the chamber, resembling the structure of myocardium (Fig. 1G)^8,21^. Chamber formation was observed in 95.93 ± 0.53% of hCdOs across different lots (Fig. 1H), and the cTnT^+^ area within the DAPI-stained region accounted for 56.45 ± 2.69% (Fig. 1I). Co-staining for cTnT and α-actinin revealed the presence of organized sarcomeric structures, including Z-discs, within the myo-like regions of hCdOs (Fig. 1J). In contrast, non-cTnT-expressing regions exhibited the expression of other cardiac constituent cells markers, including the fibroblast marker vimentin (VIM) and the smooth muscle cell marker α-smooth muscle actin (αSMA) (Fig. 1K)^8,21,22^. These findings demonstrate that the established differentiation protocol enables the reproducible generation of high-quality hCdOs, which are structurally and functionally suitable for drug responsiveness evaluation. To ensure the reliability of drug evaluation using reproducibly generated hCdOs, organoids with an average diameter of 2.5–3.0 mm were selected for assessment.

### Application of 3D-Net for measurement of electrophysiological signal in MEA chip

**Fig. 2:**
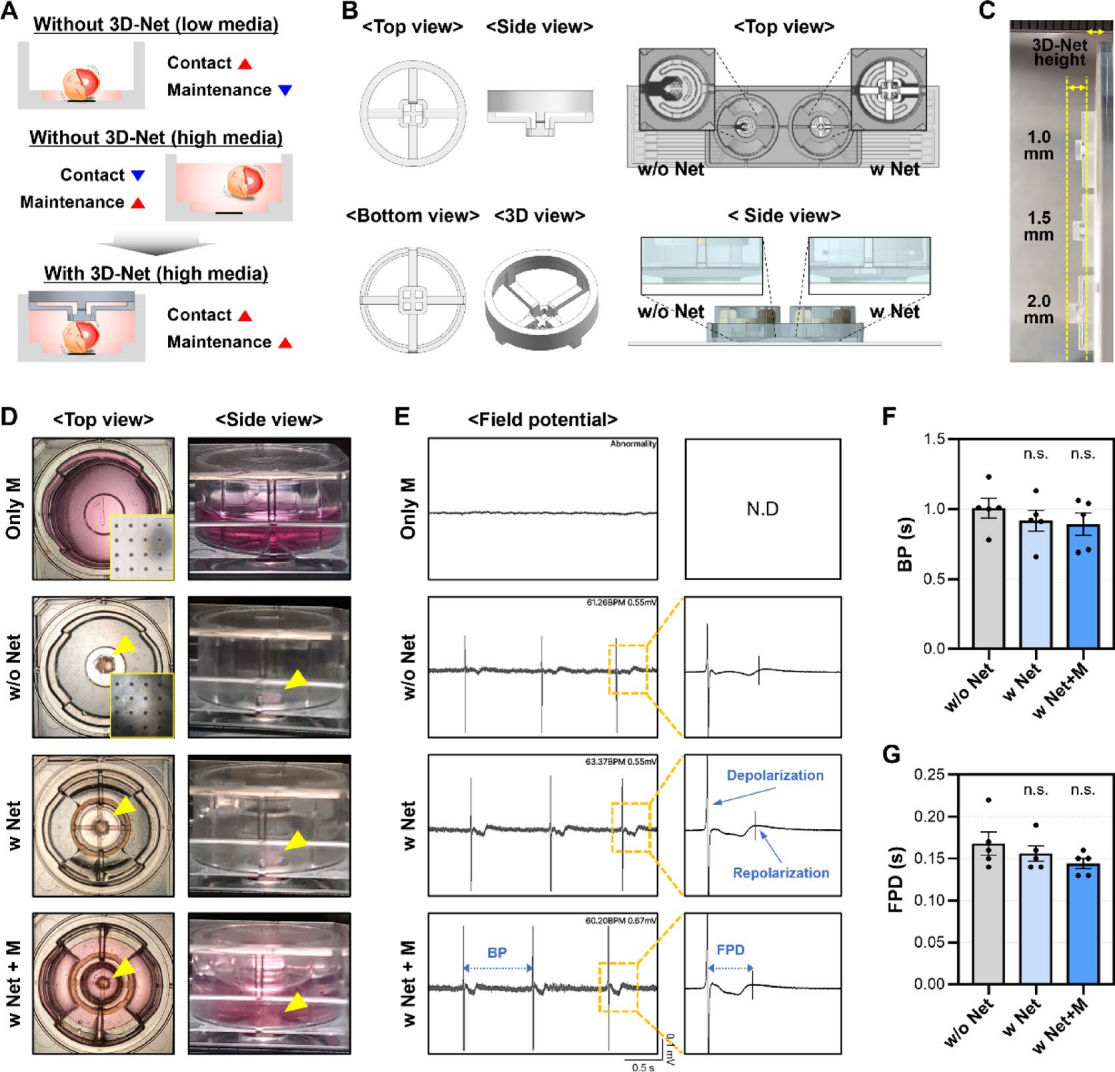
3D-Net–based measurement of MEA electrophysiological signals. (**A**) Schematic illustration for stable electrophysiological signal of hCdO. (**B**) Multiview images of 3D-Net and its integration with the MEA chip. (**C**) Comparison of 3D-Nets with different heights. (**D**) Top and side view images of only media, without 3D-Net (w/o Net), with 3D-Net (w Net), and with 3D-Net plus media (w Net + M) groups. (**E**) Representative FP raw waveforms from each group. N.D: not detected. BP: beat period. FPD: field potential duration. Comparison of change in (**F**) BP and (**G**) FPD across groups (*N* = 5). Data are expressed as the mean ± SEM. n.s.: not significant.

To achieve signal acquisition from organoids on a MEA chip, stable contact between organoids and electrodes must be maintained while preventing flotation^8,16^. A commonly used method involves using a minimal volume of culture medium to keep organoids in place, enhancing contact with electrodes. However, this volume is often insufficient to maintain viability beyond acute assessment periods (< 2 h) and limits compound dilution for drug testing^23^. In contrast, a larger medium volume improves viability and enables effective drug application but causes organoids to float, disrupting electrode contact. This problem is especially significant for spontaneously beating hCdOs, where stable contact is difficult to maintain. To address these limitations, a silicon-based 3D-Net structure was designed to physically restrict organoid movement while preserving functionality (Fig. 2A). The mesh-like structure was positioned above the electrode area to maintain organoid placement (Fig. 2B). Considering the diameter of the selected hCdOs and their contact with the electrodes, the distance from the upper mold to the organoid contact surface (3D-Net height) was set and fabricated at 1.0, 1.5, or 2.0 mm (Fig. 2B and C, and Fig. S1).

Four experimental conditions were established. The “Only M” group contained only medium without organoids. The “w/o Net” group involved organoids cultured in 50–60 µl of medium without the 3D-Net. The “w Net” group included organoids placed under the 3D-Net. The “w Net + M” group included the 3D-Net structure along with an additional 1 ml of culture medium (Fig. 2D). In the Net + M group, application of the 1.0 mm 3D-Net caused the organoids to detach from the electrodes upon media addition (Figs. 2C and Fig. S2), making it difficult to record stable electrical signals. Therefore, this height condition was excluded from further analysis, and a 1.5 mm 3D-Net, which maintained organoid contact with the electrodes even after media addition, was used for subsequent experiments (Fig. 2C and D and Fig. S2). As a result, in the w Net + M group, organoids maintained spontaneous beating while remaining in stable contact with the electrodes, despite the increased medium volume (Fig. 2D). Field potential (FP) raw waveform showed only background signals in the Only M group. In contrast, FP waveforms reflecting depolarization and repolarization were observed in the w/o Net, w Net, and w Net + M groups (Fig. 2E). Comparable raw waveform patterns were observed among the w/o Net, w Net, and w Net + M groups (Fig. 2E), with no significant differences in beat period (BP) or field potential duration (FPD) (Fig. 2F and G). Overall, the 3D-Net structure preserved the electrophysiological properties of hCdOs while preventing flotation. Next, the effect of medium volume on MEA recordings was assessed by monitoring time-dependent changes under w/o Net and w Net + M conditions. In the w/o Net, FP signals were detectable for 1 h but gradually diminished by 6 h as the organoids drifted away from the electrodes due to medium evaporation. In contrast, under w Net + M, spontaneous beating and FP signals remained comparable to baseline up to 6 h. Even at 24 h, depolarization signals were still observed with moderate amplitude reduction, which is considered to result from hCdOs being maintained in static contact with the planar electrodes for an extended period, unlike their original floating culture conditions (Fig. S3). This configuration enables signal acquisition beyond acute assessment periods and stable drug application, providing a reliable platform for organoid-based pharmacological evaluation.

### Comparison of hCdO FP signals according to 3D-Net height

**Fig. 3:**
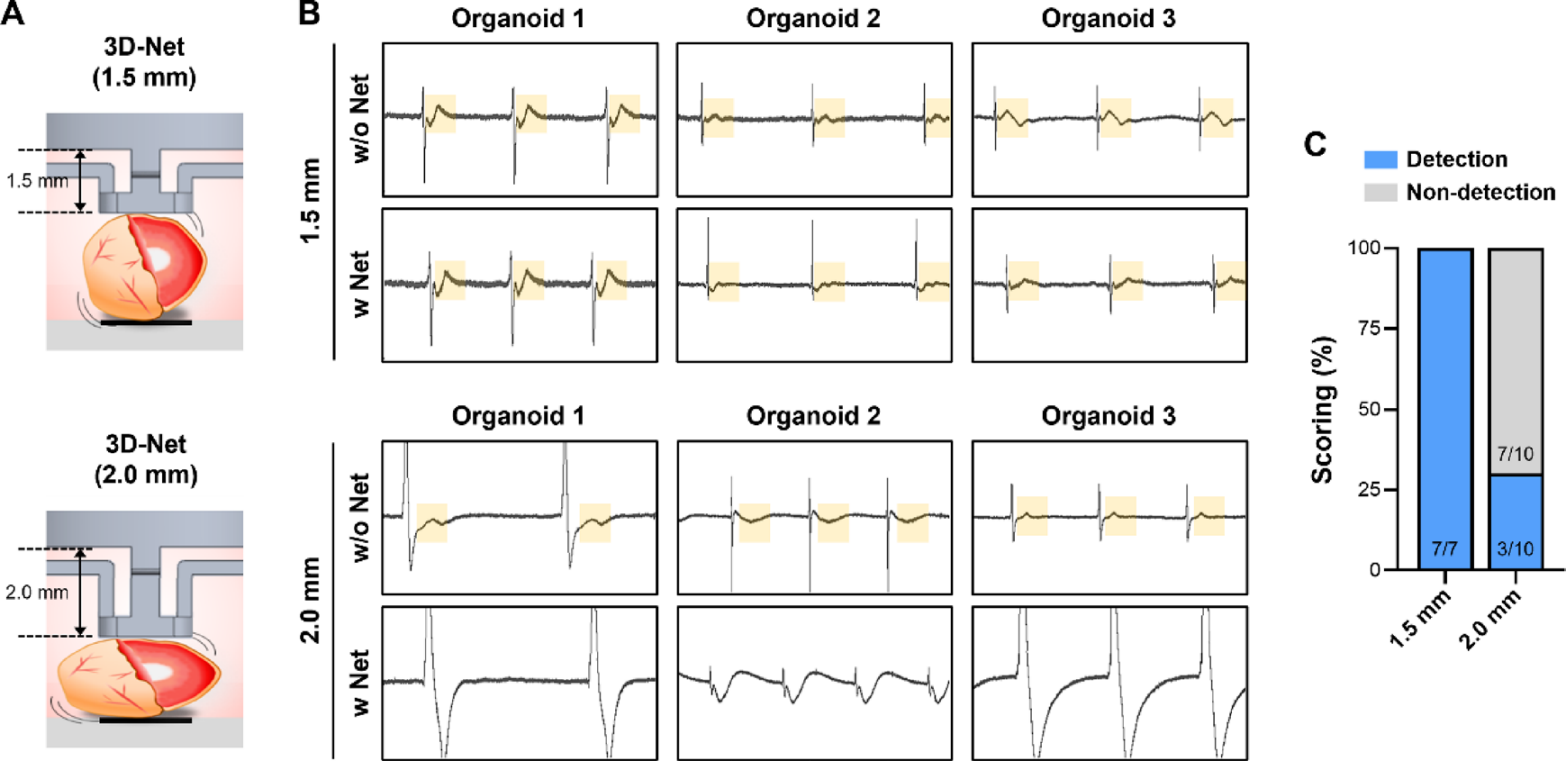
Detection of hCdO repolarization (T-wave) changes according to 3D-Net height. (**A**) Schematic images showing morphological changes of hCdO contacted on 3D-Nets with heights of 1.5 mm and 2.0 mm. (**B**) Representative FP raw waveforms reflected T-wave changes with or without the two height groups. Yellow box: T-wave region. (**C**) Comparison of normal T-wave detection scoring between groups (1.5 mm, *N* = 7; 2.0 mm, *N* = 10). Data are expressed as the mean ± SEM.

To evaluate the effect of physical pressure between the electrodes and organoids on hCdO FP signals, 1.5 mm and 2.0 mm 3D-Nets were applied (Fig. 3A). As shown in Fig. 2E, application of the 1.5 mm 3D-Net maintained regular depolarization and repolarization, similar to the signals observed before 3D-Net application (Fig. 3B). In contrast, the 2.0 mm 3D-Net induced waveform distortions and failure to detect T-waves, which were not observed previously (Fig. 3B). Analysis of T-wave detection scoring in the FP signals showed that 100% of T-waves were preserved in all organoids with the 1.5 mm 3D-Net, whereas only 30% were detected with the 2.0 mm 3D-Net (Fig. 3C). These results demonstrate that an inappropriate 3D-Net height can exert excessive physical pressure on hCdOs, disrupting normal electrophysiological signals. In addition to electrode-based FP measurements, imaging-based calcium transient analysis was used to compare the same hCdOs under 1.5 mm and 2.0 mm 3D-Nets. Compared with the signals observed with the 1.5 mm 3D-Net, replacing it with a 2.0 mm 3D-Net resulted in prolonged F_peak_ intervals and decreased calcium amplitudes (ΔF/F_0_) (Fig. S4). Therefore, drug screening was performed using the 1.5 mm 3D-Net, which provided stable signal recordings.

### Drug screening using 3D Net-assisted platform

**Fig. 4:**
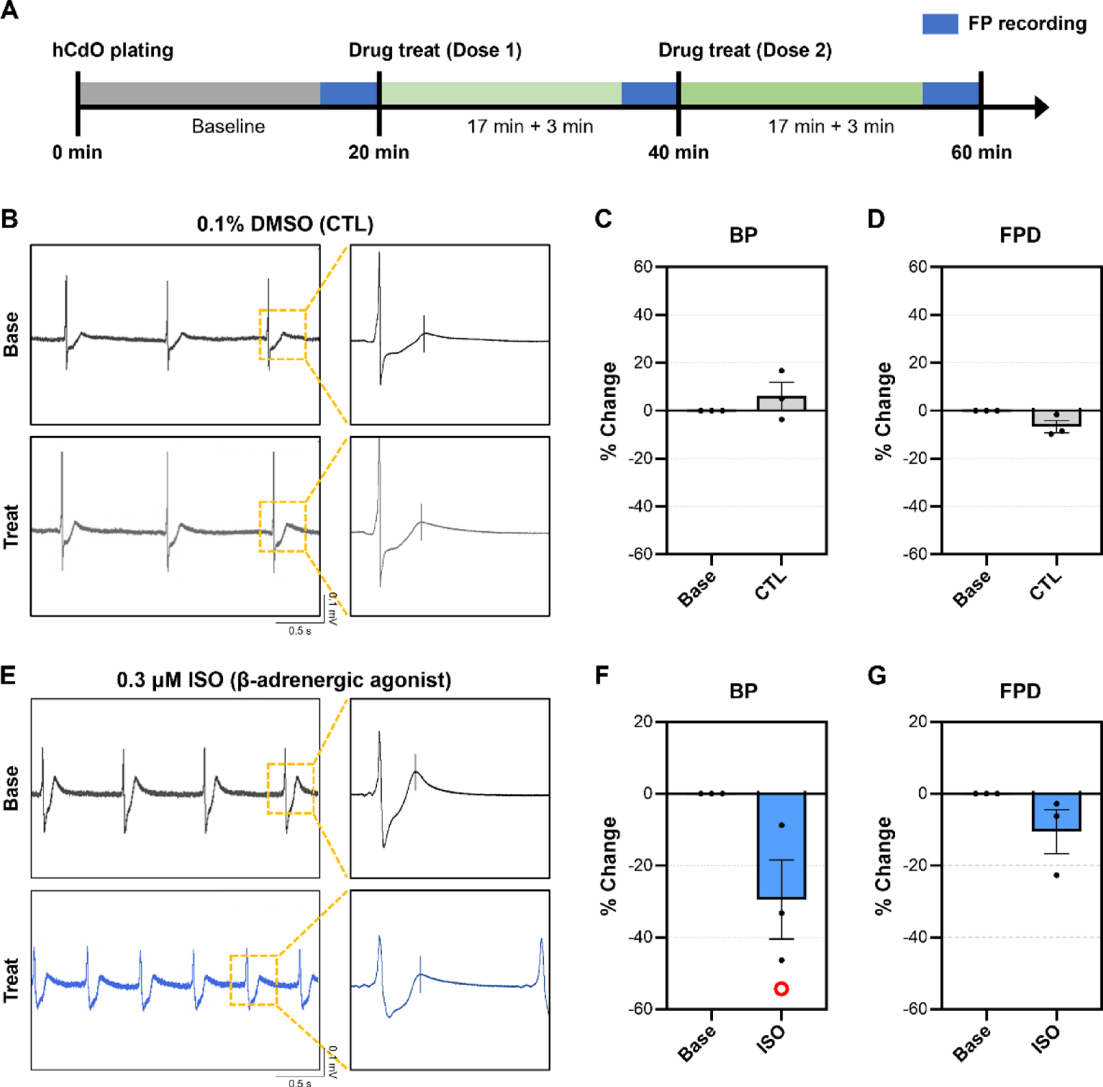
Evaluation of control and the β-adrenergic agonist isoproterenol using 3D-Net. (**A**) Protocol for recording FP and assessing drug responsiveness on a MEA chip using the 3D-Net. (**B**) Representative FP raw waveforms before (Baseline, Base) and after treatment (Treat) with 0.1% DMSO (CTL). Changes in (**C**) BP and (**D**) FPD following CTL treatment (*N* = 3). (**E**) Representative FP raw waveforms at base and after treatment with 0.3 µM isoproterenol (ISO). Changes in (**F**) BP and (**G**) FPD following 0.3 µM ISO treatment (*N* = 3). Data are expressed as the mean ± SEM. O: Notation for changes of more than 20%.

Drug evaluation was conducted under w Net + M condition following the plating of hCdOs (Fig. 4A). To verify proper establishment of the 3D-Net-based platform for evaluating drug, initial assessments were conducted under control (CTL). The CTL group was treated with 0.1% DMSO, a dose known not to induce cytotoxicity^6^. As a result, no noticeable differences were observed in the raw waveform compared to the baseline (Base), and changes in both BP (6.07 ± 5.09%) and FPD (-6.61 ± 2.55%) were negligible (Fig. 4B and D, and Video S1). Isoproterenol (ISO), a sympathomimetic agent and known β-adrenergic receptor agonist, has been reported to enhance cardiac beating activity^24^. Treatment of hCdO with 0.3 µM ISO significantly decreased the BP by 29.47 ± 11.02%, which was consistent with an increased number of depolarization events observed in the raw waveform compared to Base (Fig. 4E and F, and Video S2). The FPD showed a slight decrease of 10.57 ± 6.12% (Fig. 4G). These results demonstrate that a method for reliably evaluating drug responses of hCdOs using the 3D-Net has been established.

### Comparison of drug responsivness between w/o net and w Net + M

**Fig. 5:**
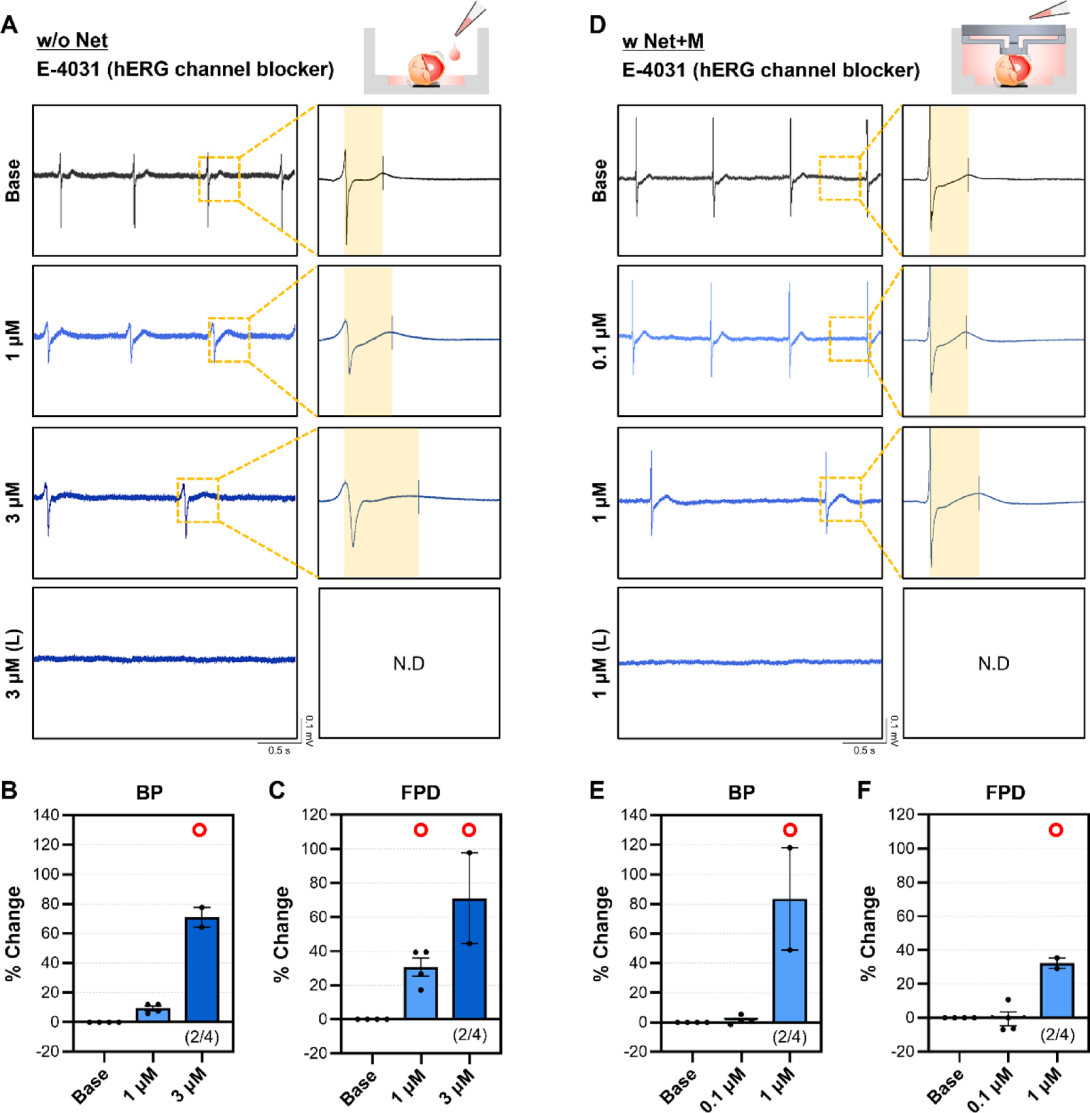
Evaluation of hERG channel blocker E-4031 between w/o Net and w Net + M. (**A**) Representative FP raw waveforms in the w/o Net condition for the Base, 1 µM E-4031, 3 µM E-4031, and 20 min after treatment with 3 µM E-4031 (L) groups. Changes in (**B**) BP and (**C**) FPD following E-4031 treatment in the w/o Net (*N* = 4). (**D**) Representative FP raw waveforms in the w Net + M condition for the Base, 0.1 µM E-4031, 1 µM E-4031, and 20 min after treatment with 1 µM E-4031 (L) groups. Changes in (**E**) BP and (**F**) FPD following E-4031 treatment in the w Net + M (*N* = 4). N.D: not detected. Data are expressed as the mean ± SEM. O: Notation for changes of more than 20%.

To evaluate the effect of the volume of culture medium on the drug responsiveness of hCdOs, E-4031 was administered under two conditions: the conventional w/o Net condition (approximately 50 µL of medium), commonly used in other MEA studies^8,25,26^, and the w Net + M condition (approximately 1 mL of medium), which represents the main focus of this study. E-4031 is an experimental class III antiarrhythmic agent that selectively blocks the voltage-gated potassium channel human Ether-à-go-go-Related Gene (hERG), which is predominantly expressed in the heart. Inhibition of the hERG channel delays repolarization of the cardiac action potential, leading to prolongation of the FPD and an increased risk of arrhythmia^6,27^. Under the w/o Net condition, treatment with 1 µM E-4031 increased BP by 9.32 ± 1.54% and significantly prolonged FPD by 30.66 ± 5.40% (Fig. 5A and C). At 3 µM, both BP (70.93 ± 6.73%) and FPD (71.14 ± 26.68%) were significantly greater than those observed with 1 µM, and the raw waveform showed a marked prolongation of the T wave (Fig. 5A and C). Within 20 min of 3 µM treatment, spontaneous beating was abolished in two hCdOs. After 20 min, most hCdOs stopped beating entirely and produced waveforms with no detectable depolarization/repolarization events (Fig. 5A and C). Under the w Net + M condition, treatment with 0.1 µM E-4031 produced minimal changes in BP (1.25 ± 1.47%) and FPD (–0.63 ± 4.10%) (Fig. 5D and F). In contrast, 1 µM E-4031 significantly prolonged BP by 83.47 ± 34.52% and FPD by 32.32 ± 3.08% (Fig. 5D and F). At the same dose of 1 µM, BP and FPD changes differed between the two conditions. BP showed more than a 70% difference in the w Net + M condition compared to the w/o Net condition (Fig. 5B and E), whereas FPD showed almost no difference (Fig. 5C and F). In addition, unlike in the w/o Net condition, all hCdOs completely stopped beating after 20 min of 1 µM E-4031 treatment (Video S3). To further evaluate the potential mechanical influence of the 3D-Net, the drug responsiveness to 3 µM E-4031 was compared between the w/o Net and w Net conditions without adding additional medium. In both conditions, treatment with 3 µM E-4031 induced a marked increase in BP and FPD compared with baseline; however, no significant differences were observed between the two groups (Fig. S5). These findings indicate that under conditions where the 3D-Net does not impose mechanical deformation on the organoids, the degree of drug dilution determined by culture medium volume can significantly influence the drug responsiveness of hCdOs. Therefore, they underscore the need to establish standardized protocols that account for various environmental factors to ensure reliable drug response assessments.

## Discussion

One of the key elements in a drug screening platform is quality control (QC) of the cells used^28^. Without proper QC, the consistency of drug responses decreases, reducing the reliability and reproducibility of the study, which ultimately results in significant waste of time and cost^28^. hCdOs are still in the early stages of research compared with hiPSC-CMs, and their characteristics can vary greatly due to differences in differentiation protocols among institutions^7–10^. Therefore, ensuring reproducibility through standardized QC based on an established differentiation protocol is essential^29^. In this study, comprehensive QC of hCdOs was performed based on a previously established *in-house* protocol. As a result, similar diameter distributions were observed among three different lots, and all lots exhibited over 90% beating efficiency, cell viability, and chamber formation rate, confirming high consistency. Furthermore, cTnT-positive cells within hCdOs were localized around the chamber periphery, similar to the myocardium, and the expression density was 56.45 ± 2.69%, showing characteristics comparable to those of the human heart (Fig. 1). To establish a reliable 3D-based cardiotoxicity assessment platform, systematic quality control criteria and a database of diverse organoids must be developed.

One of the key findings of this study is that excessive physical pressure applied to organoids can lead to the collapse of electrophysiological signals. When a 1.5 mm 3D-Net was applied to hCdOs with diameters of 2.5–3.0 mm, selected through QC, normal depolarization and repolarization coupling was preserved. In contrast, applying a 2.0 mm 3D-Net exerted excessive physical pressure on the hCdOs, effectively preventing their floating but failing to maintain the integrity of their electrophysiological signals (Fig. 3). Notably, failure to detect T-waves under such pressure prevents measurement of the QT interval, the most critical indicator in cardiotoxicity assessment, making these conditions unsuitable for evaluation. Previous studies have also reported the mechanical activity of CM aggregates under mechanical compression^30^. Compression applied to CM aggregates on parallel plates tended to enhance and stabilize synchronized beating, but excessive pressure induced irregularities and transient cardiac arrest. These findings indicate that establishing an appropriate range of mechanical pressure is a critical control parameter when evaluating electrophysiological signals in cardiac tissue constructs. Another key finding of this study is that the volume of culture medium, which determines the degree of drug dilution, caused marked variability in the electrophysiological responses of hCdOs. In the w/o Net condition, cardiac arrest occurred only after treatment with 3 µM E-4031. In contrast, in the w Net + M condition, treatment with 1 µM E-4031 for more than 20 min was sufficient to completely stop beating. At the same dose of 1 µM, BP differed by more than 70% between the two conditions (Fig. 5). This indicates that a smaller medium volume is associated with reduced drug responsiveness in hCdOs. These results show that precise standardization of experimental parameters is essential for generating reproducible and interpretable drug response data^31,32^. The importance of such standardization is even greater in specialized preclinical testing environments, such as contract research organization (CRO). In these settings, strict control and clear definition of environmental factors are critical to producing results that are both scientifically sound and applicable to clinical or regulatory decision-making^32,33^.

Despite utilizing organoids that exhibit high reproducibility and incorporate 3D complexity, the conventional 2D MEA chip remains limited in that it can only capture electrophysiological signals at points of direct contact with the organoid^8,34^. Furthermore, the silicon-based 3D-Net employed in this study was designed for organoids within a specific diameter range (Fig. 2B and C), necessitating further evaluation of its applicability to organoids of varying sizes produced by other research groups^9,10^. As our results indicate (Figs. 3 and 5), a critical factor for 3D sensing is that the sensor must contact the organoid without exerting excessive physical pressure to ensure stable and accurate signal acquisition. In addition, proper standardization of the medium volume within the wells of the chip is essential to enable appropriate screening of drug response doses. Therefore, future efforts should focus on the development of flexible sensing platforms capable of supporting organoids without mechanical damage while allowing simultaneous signal acquisition from multiple regions, alongside standardization of 3D platforms to ensure broader applicability^16,18,35,36^. Nevertheless, the integration of the proposed 3D-Net with a 2D MEA chip in this study successfully enabled stable acquisition of drug response data from hCdOs, providing a robust foundation for the advancement of next-generation, organoid-based 3D drug evaluation platforms.

## Materials & methods

### Generation of human induced pluripotent stem cells (hiPSCs) derived cardiac organoids (hCdOs)

hiPSCs were generated by reprogramming BJ fibroblasts (ATCC) using episomal vectors, as previously described^37^. hiPSCs were seeded on Matrigel (Corning Inc.)-coated dishes in mTeSR™1 medium (STEMCELL Technologies Inc., Vancouver, BC, Canada) supplemented with 10 µM Y-27,632 (Tocris Bioscience). Medium was changed daily until ~ 90% confluency. hiPSCs were detached using DPBS containing 0.5 mM EDTA, collected in mTeSR™1 medium with 10% Matrigel and 10 µM Y-27,632 (Tocris Bioscience), and plated onto 60-mm petri dishes. To generate embryonic bodies (EBs), the medium was changed daily and cultures were maintained on a shaker inside an incubator for 3 days. The EBs were then cultured for 2 days in RPMI1640 (Gibco) supplemented with B27 without insulin (B27(-); Gibco) and 6 µM CHIR99021 (Tocris Bioscience). This was followed by an additional 2 days of culture in RPMI1640 + B27(−) supplemented with 2 µM C-59 (Selleckchem). From day 7 of differentiation, the medium was replaced with Advanced MEM (AD-MEM; Gibco) supplemented with 1% GlutaMAX (Gibco) and penicillin/streptomycin (Gibco), and changed every other day. Spontaneous beating of differentiated hCdOs was observed around day 11, and samples were collected on day 20 for analysis. This differentiation protocol was adapted from a previously established method (Fig. 1A)^8^.

### Live/Dead assay

Cell viability was evaluated using the LIVE/DEAD^®^ Viability/Cytotoxicity Kit (Invitrogen). hCdOs were washed with PBS and incubated for 30 min in AD-MEM containing 2 µM Calcein AM and 4 µM EthD-1. Fluorescence was detected using FITC (C-AM) and TRITC (EthD-1) channels under a fluorescence microscope. Image J was used to quantify fluorescence, and the ratio of C-AM to EthD-1 was calculated.

### Immunocytochemistry (ICC)

D20 hCdOs were fixed with 4% PFA at 4 °C. Fixed hCdOs were incubated 15% and 30% sucrose solution for 1 day each, then embedded in OCT compound. Cryosections (7–9 μm) were prepared using a cryostat at − 20 °C. Cryosections were permeabilized and blocked in PBS containing 0.1% Triton X-100 (Sigma-Aldrich) and 3% normal goat serum (NGS; Thermo Fisher Scientific) for 30 min at RT. Samples were incubated overnight at 4 °C with primary antibodies (1:200) against cTnT (Abcam, ab45932), α-actinin (Sigma-Aldrich, A7811), anti-vimentin (VIM; Abcam, ab8978), and anti-α-smooth muscle actin (αSMA; Abcam, ab5694). After washing, samples were incubated for 2 h at RT with secondary antibodies (Alexa Fluor 488 goat anti-mouse IgG and Alexa Fluor 594 goat anti-rabbit IgG (1:700; Invitrogen)). Nuclei were counterstained with DAPI (1:1000; Thermo Fisher Scientific). Fluorescence images were acquired using a Nikon TE2000-U microscope (Nikon) and fluorescence intensities quantified using Image J. The colors were adjusted and indicated for clear distinction.

### Preparation of chemicals

Isoproterenol (Sigma-Aldrich) and E-4031 (Sigma-Aldrich) were dissolved in dimethyl sulfoxide (DMSO; Sigma-Aldrich). Each drug was prepared at 10× the working dose in DMSO and diluted in AD-MEM before use. The final DMSO dose was kept below 0.1%, which is considered non-cytotoxic.

### Electrophysiological signal analysis in microelectrode array (MEA) using 3D-Net

To effectively restrict the movement of organoids in a large-volume medium environment, a 3D-Net (Cellames) was fabricated. The 3D-Net was fabricated from a biocompatible polydimethylsiloxane (PDMS) based silicone material. The height of the 3D-Net was adjusted to 1.0 mm, 1.5 mm, or 2.0 mm based on the diameter of the organoids, thereby controlling the distance between the bottom surface and the comb-like structure. Only the portion of the 3D-Net in contact with the organoids was varied in height, while all other dimensions were kept constant. Detailed specifications are shown in Fig. S1. To perform measurements without the use of 3D-Net, hCdOs were placed at the center of the well containing electrodes in 16E-CFPS Chip (Cellames), followed by the addition of 50 µl of AD-MEM. For field potential (FP) analysis, chip was inserted in to the CFPS-32 device (Cellames) within a CO_2_ incubator. After 17 min stabilization, baseline (Base) FP signal was recorded for 3 min. Next, to record signals with the 3D-Net, the 3D-Net was carefully inserted to align with the comb structure, ensuring that the organoid was positioned at the center of 3D-Net. Additionally, 1 ml AD-MEM medium was gently added to prevent movement of the organoid. Signals for each group were recorded in the same manner as previously described. For drug evaluation, 5 µl (without 3D-Net) or 100 µl (with 3D-Net + media) of medium was removed from the chip and replaced with 10× drugs solution. This method was applied with reference to previous CiPA reports^5,27^. For all drug evaluations, signals were recorded for 3 min after 17 min of incubation following drug treatment. The recorded FP raw waveforms were analyzed using the CFPS-32 software.

### Calcium transient imaging

To compare calcium transients of hCdO according to the heigt of 3D-Net, the hCdO was incubated with 5 µM Fluo-4 AM (Invitrogen) and 0.02% Pluronic F-127 for 1 h. The hCdO was then washed once with fresh culture medium. Fluorescence signals were acquired using a Nikon TE2000-U microscope in the FITC channel, during which videos were recorded. For the same hCdO, approximately 1 min recording was first performed after applying the 1.5 mm 3D-Net, followed by an immediate replacement with the 2.0 mm 3D-Net and an additional 1 min recording. The acquired images were analyzed in ImageJ by selecting regions of interest (ROI) and extracting frame-by-frame fluorescence intensity. Calcium amplitude over time was quantified as ΔF/F_0_, where F represents the fluorescence intensity at each frame and F_0_ is defined as the baseline fluorescence intensity within the ROI across all frames.

### Statistical analysis

Quantitative data are presented as mean ± standard error of the mean (SEM). ‘N’ refers to (1) independently prepared dishes (lots) used for hCdOs generation, or (2) independent experimental sets using individual hCdOs measured in separate wells of MEA chip. ‘n’ indicates the number of distinct organoids measured within a single dish. All experiments were performed in at least three independent studies, and the corresponding values of N and n for each experiment are provided in the figure legends. Statistical analysis was performed by one-way ANOVA with Tukey’s test and two-way ANOVA. A p value (*p < 0.05, **p < 0.01, and ***p < 0.001) was considered statistically significant. Data graphs were created using GraphPad Prism version 10.4.2. Based on CiPA-related reports, an FPDc increase of over 20% may be considered indicative of a proarrhythmic risk^5,38,39^. Accordingly, a > 20% change was set as the criterion for significance and marked as O.

## Supplementary Information

Below is the link to the electronic supplementary material.

Supplementary Material 1

Supplementary Material 2

Supplementary Material 3

Supplementary Material 4

## Data Availability

All data generated or analyzed during this study are included in this published article.
