# Supplementary material for: Non-Invasive electrophysiological monitoring of cardiac organoids using 3D-Net-assisted microelectrodes array platform
Source: Sci Rep. 2026 Jan 24;16:4428. doi: 10.1038/s41598-025-34504-3 (PMC12865037; doi:10.1038/s41598-025-34504-3)
Supplement: Supplementary file 1 — Supplementary Material 1 [file 41598_2025_34504_MOESM1_ESM.docx]

**Supplementary information**

**Non-Invasive Electrophysiological Monitoring of Cardiac Organoids using 3D-Net-Assisted Microelectrodes Array Platform**

Shinhye Park^1, &^, Sang-Jun Cho^2^, C-Yoon Kim^1^, Hyung Min Chung^3^, and Seul-Gi Lee^1, *^

^1^ College of Veterinary Medicine, Konkuk University, Seoul, 05029, Republic of Korea

^2^ Cellames Inc., 405, 19 Wiryegwangjang‑ro, Sujeong‑gu, Seongnam‑si, Gyeonggi‑do, Republic of Korea

^3^ Department of Stem Cell Biology, School of Medicine, Konkuk University, 120 Neungdong-Ro, Gwangjin-Gu, Seoul Republic of Korea, 05029

^&^ This author contributed to this work as first author.

^*^ This author contributed to this work as corresponding author.

*** Corresponding Author**

: Seul-Gi Lee

College of Veterinary Medicine, Konkuk University, Seoul, 05029, Republic of Korea.; E-mail: maxwisdom@konkuk.ac.kr


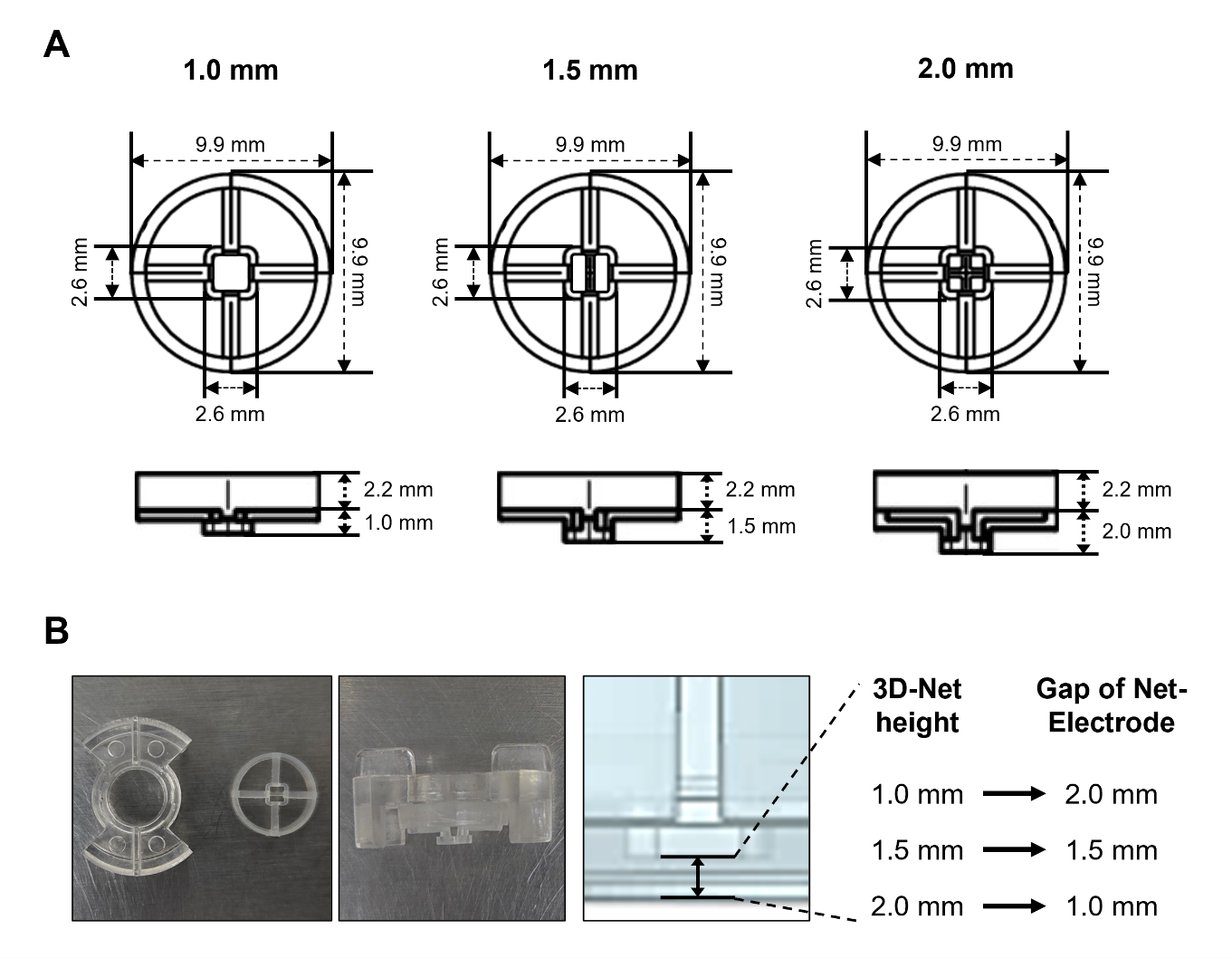


**Fig. S1. Detailed dimensions of the 3D-Net.** (A) Comparison of the detailed dimensions among the 1.0, 1.5, and 2.0 mm 3D-Nets. (B) Distance between the 3D-Net and the electrode area within the MEA chip.


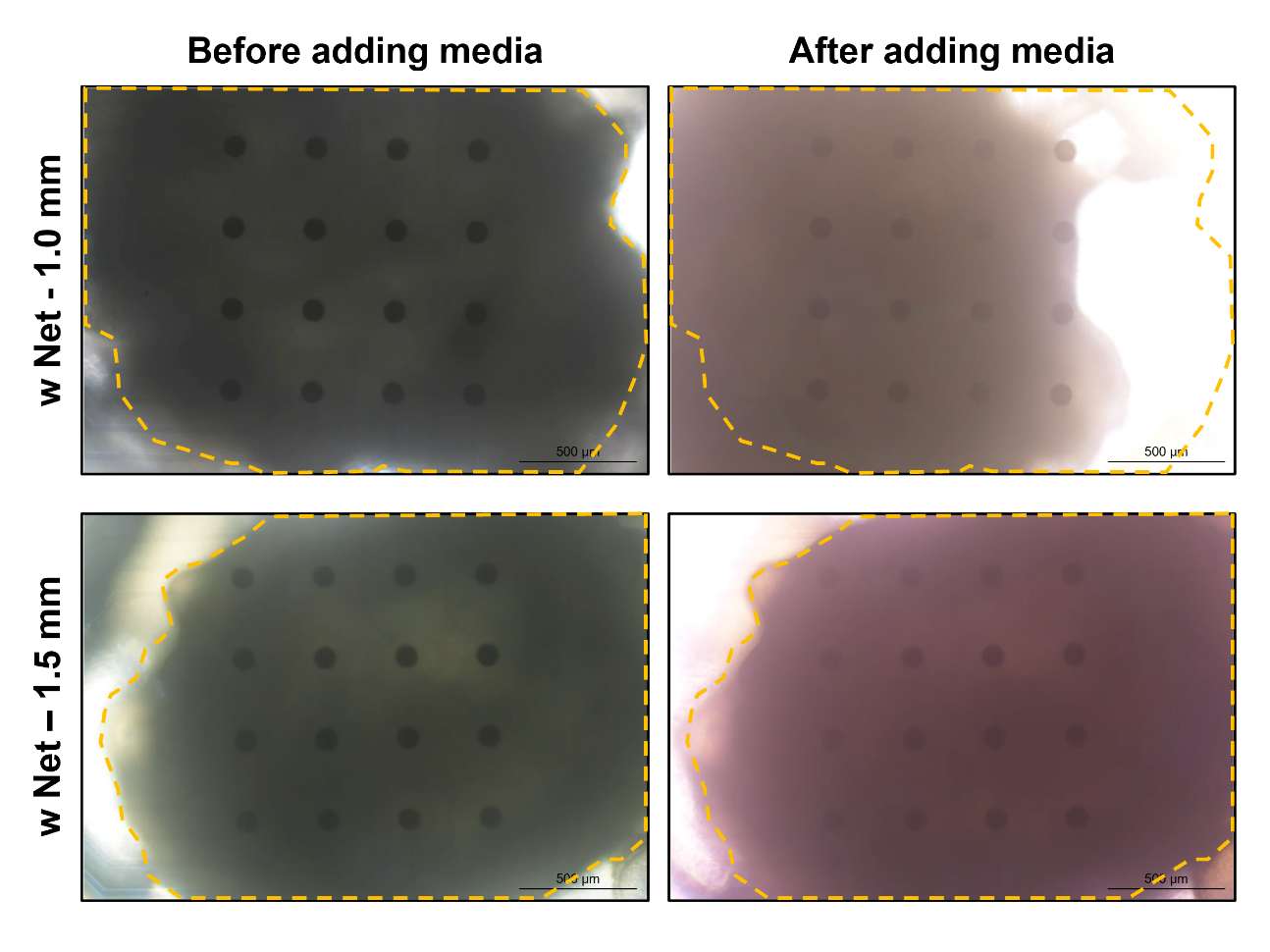


**Fig. S2. Comparison of hCdOs displacement upon culture media addition after applying the 1.0 mm and 1.5 mm 3D-Nets.** Yellow dotted line: hCdO boundary before media addition. Scale bar: 500 μm.


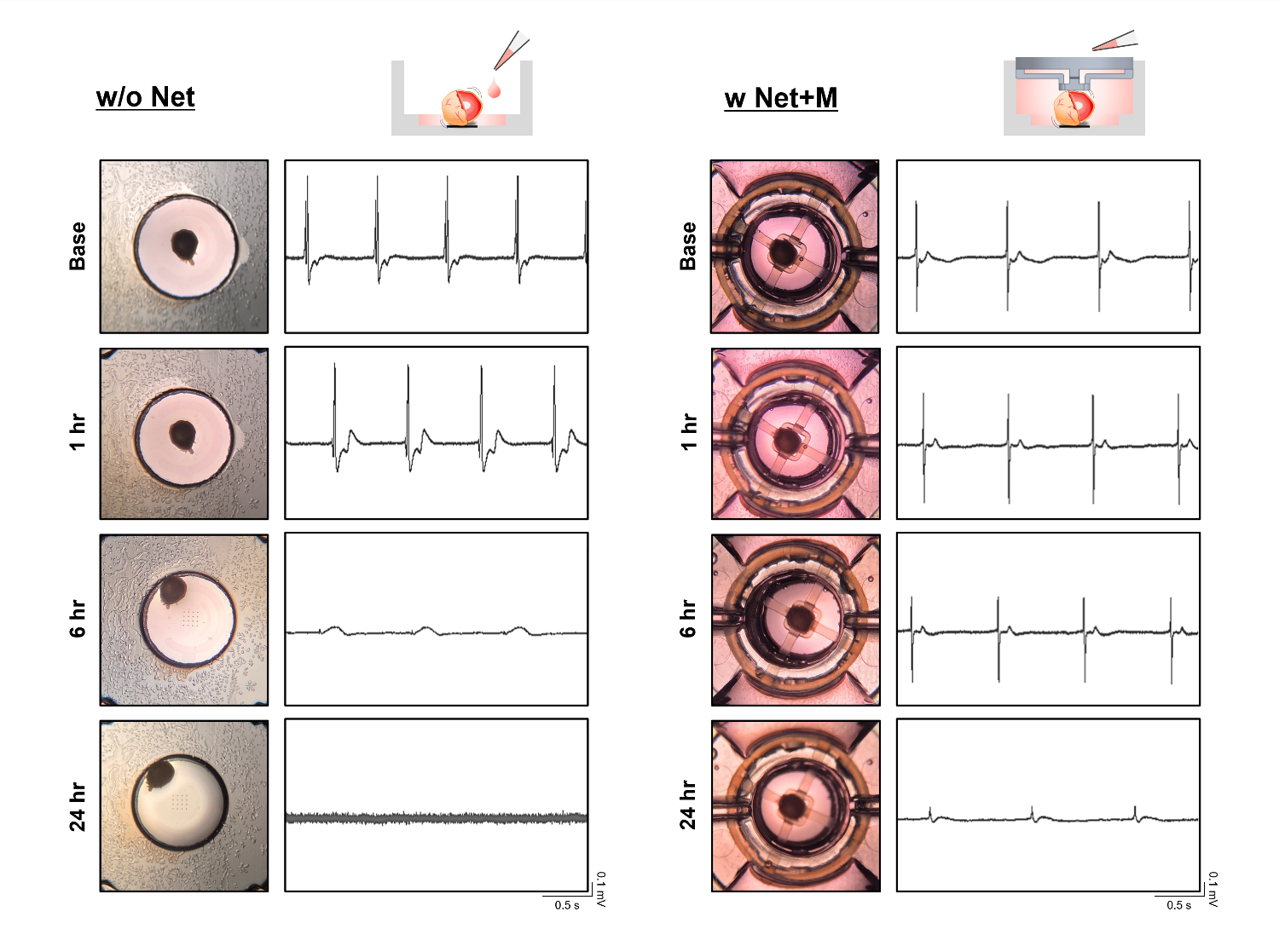


**Fig. S3. Comparison of time-lapse MEA analysis between the w/o Net and w Net+M conditions.** Comparison of the morphology and FP raw waveforms of hCdOs within the MEA chip at Base (30 min), 1 hr, 6 hr, and 24 hr after hCdO loading.


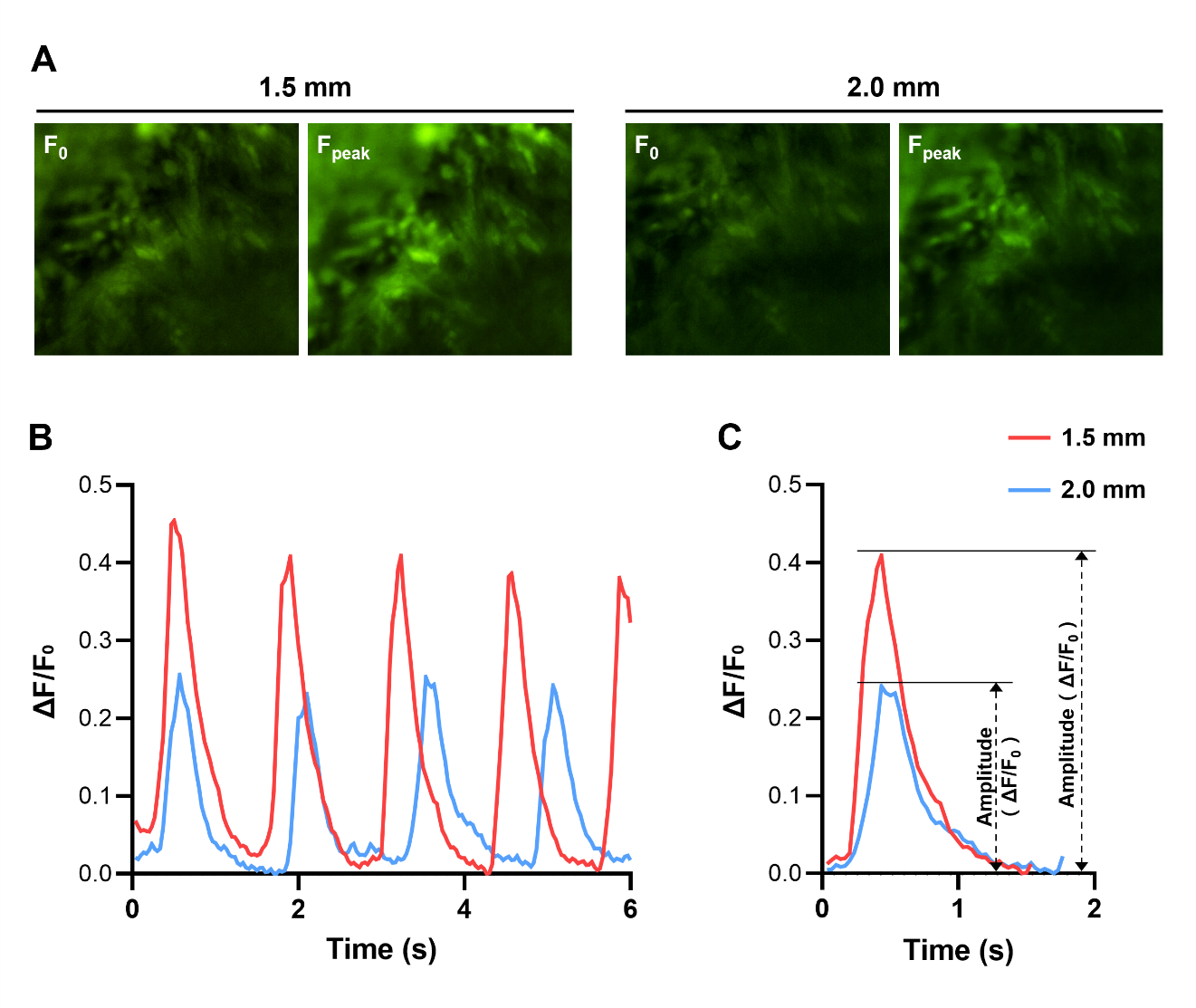


**Fig. S4.** **Comparison of calcium imaging between the 1.5 mm and 2.0 mm 3D-Nets.** (A) Representative calcium fluorescence images (F₀ and F_peak) obtained from the same hCdO after sequential application of the 1.5 mm and subsequently the 2.0 mm 3D-Net. (B) Comparison of raw calcium transient imaging waveforms between the two conditions. (C) Comparison of calcium amplitude (ΔF/F_0_) based on single-peak waveform.


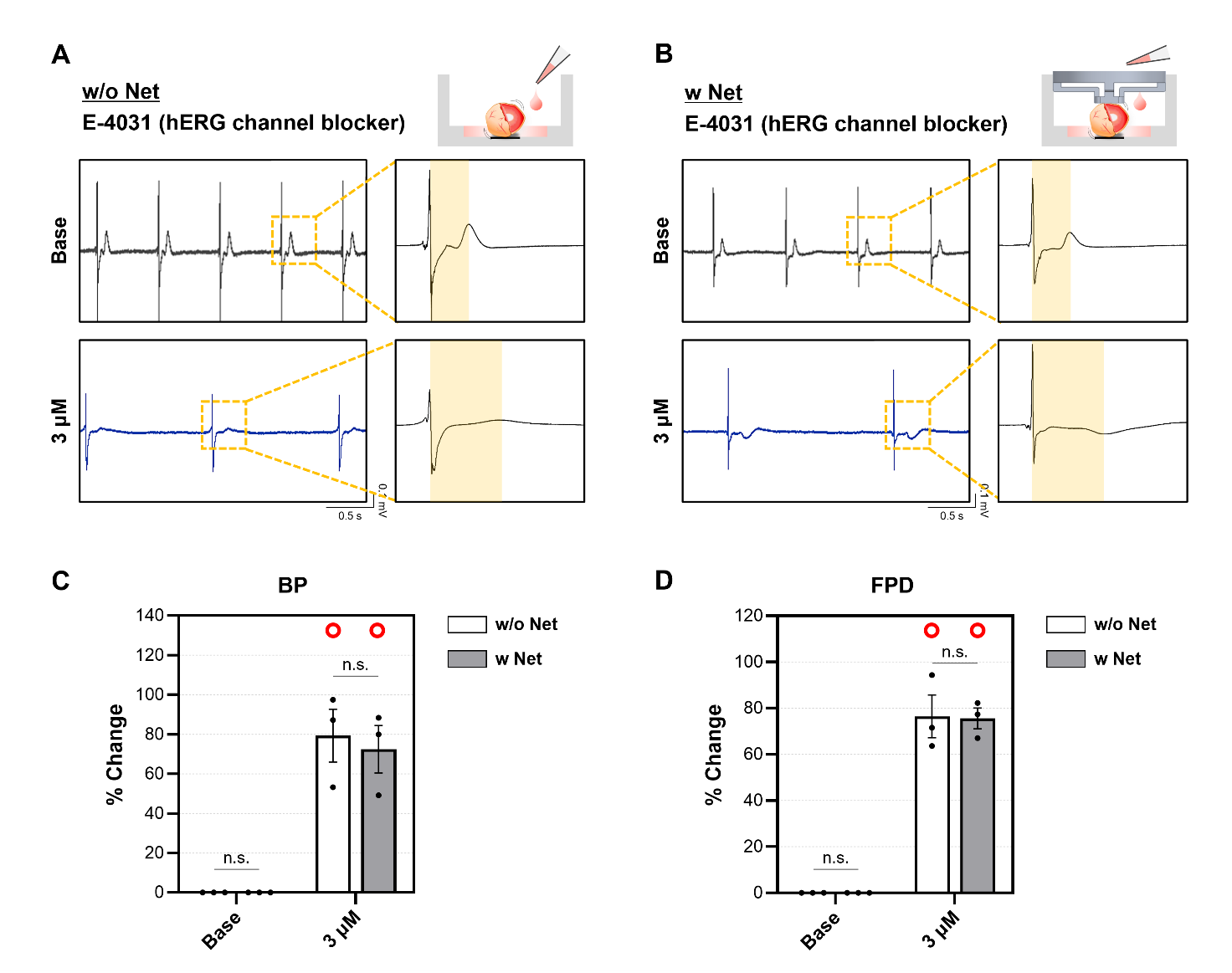


**Fig. S5. Evaluation of hERG channel blocker E-4031 between w/o Net and w Net.** (A and B) Representative FP raw waveforms for the Base and 3 µM E-4031 treatment groups under the w/o Net and w Net conditions. (C and D) Comparison of BP and FPD changes among groups following 3 µM E-4031 treatment (N=3). Data are expressed as the mean ± SEM. n.s.: not significant. O: Notation for changes of more than 20%.
